# Identification and characterization of short-chain dehydrogenase/reductase 3 (DHRS3) deficiency, a retinoic acid embryopathy of humans

**DOI:** 10.1016/j.gimo.2025.103427

**Published:** 2025-03-29

**Authors:** Akiko Soneda Hashimoto, Jianshi Yu, Christina Williams, Karin Gaudenz, Parisa Varshosaz, Ruonan Zhao, Nageswara Pilli, Tian Liu, Jonathon Russell, Rebecca S. Tooze, Stephen R.F. Twigg, Siddharth Banka, Elizabeth Sweeney, Simon J. McGowan, Samantha J.L. Knight, Jenny C. Taylor, Tawfiq Jamal Froukh, M. Irene Valenzuela Palafoll, Núria Martínez-Gil, Mar Costa-Roger, Maria Teresa Villarreal-Molina, Esther Lieberman Hernandez, Rami Abou Jamra, Felix Gattermann, Margarete Koch-Hogrebe, Dagmar Wieczorek, Paul A. Trainor, Alexander R. Moise, Andrew O.M. Wilkie, Maureen A. Kane

**Affiliations:** 1 Clinical Genetics Group, MRC Weatherall Institute of Molecular Medicine, University of Oxford, Oxford, United Kingdom; 2 Department of Pharmaceutical Sciences, University of Maryland School of Pharmacy, Baltimore, MD; 3 Stowers Institute for Medical Research, Kansas City, MO; 4 Medical Sciences Division, Northern Ontario School of Medicine University, Sudbury, ON, Canada; 5 Oxford NIHR Biomedical Research Centre, Centre for Human Genetics, University of Oxford, Oxford, United Kingdom; 6 Division of Evolution, Infection and Genomics, School of Biological Sciences, Faculty of Biology, Medicine and Health, University of Manchester, Manchester, United Kingdom; 7 Manchester Centre for Genomic Medicine, St Mary’s Hospital, Manchester University NHS Foundation Trust, Health Innovation Manchester, Manchester, United Kingdom; 8 Department of Clinical Genetics, Liverpool Women's NHS Foundation Trust, Liverpool, United Kingdom; 9 Centre for Computational Biology, MRC Weatherall Institute of Molecular Medicine, University of Oxford, Oxford, United Kingdom; 10 Department of Biotechnology and Genetic Engineering, Philadelphia University, Amman, Jordan; 11 Department of Clinical and Molecular Genetics, Vall d´Hebron University Hospital, Barcelona, Spain; 12 Medicine Genetics Group, Vall d’Hebron Research Institute, Barcelona, Spain; 13 Laboratorio de Genómica Cardiovascular, Instituto Nacional de Medicina Genómica, Mexico City, Mexico; 14 Departamento de Genética Humana, Instituto Nacional de Pediatría, Mexico City, Mexico; 15 Institute of Human Genetics, University of Leipzig Medical Center, Leipzig, Germany; 16 Institute of Human Genetics, Medical Faculty and University Hospital Düsseldorf, Heinrich Heine University Düsseldorf, Düsseldorf, Germany; 17 Vestische Kinder und Jugendklinik Datteln, Universität Witten-Herdecke, Datteln, Germany; 18 University of Kansas Medical Center, Department of Anatomy and Cell Biology, Kansas City, KS

**Keywords:** 5′-untranslated region, Craniosynostosis, Noncoding variant, Retinoic acid, Vitamin A

## Abstract

**Purpose:**

Signaling by the morphogen all-trans retinoic acid (RA) is critical for embryonic development, during which its tissue concentration must be tightly regulated. We investigated 8 sibships (12 individuals) segregating 5 different homozygous variants of dehydrogenase/reductase 3 **(***DHRS3*), which encodes an embryonically expressed enzyme (short-chain dehydrogenase/reductase 3; also termed SDR16C1) that catalyzes the reduction of retinaldehyde to retinol, limiting excessive RA synthesis.

**Methods:**

We assessed variant pathogenicity using comparative phenotypic and bioinformatic analysis, quantification of *DHRS3* expression, and measurement of plasma retinoid metabolites.

**Results:**

Five homozygotes from 3 families (1 family segregating a deletion of the promoter and 5′-untranslated region of *DHRS3*, the other 2 a missense variant p.(Val171Met)), manifested a congruent phenotype, including coronal craniosynostosis, dysmorphic facial features, congenital heart disease (4/5 individuals), and scoliosis (5/5 individuals). Transcription of *DHRS3* in whole blood cells from 2 homozygotes for the promoter/5′-untranslated region deletion was 90% to 98% reduced. Cells transfected with a DHRS3-Val171Met construct exhibited reduced retinaldehyde reduction capacity compared with wild-type, yielding reduced retinol and elevated RA; correspondingly, plasma from homozygous patients had significantly reduced retinol and elevated RA (exceeding the normal range), compared with controls and heterozygous relatives. Three additional homozygous missense variants of DHRS3 (p.(Val110Ile), p.(Gly115Asp), and p.(Glu244Gln)) were shown to reduce catalytic activity in vitro and/or in vivo but were associated with normal or different phenotypes that did not meet the threshold to assign likely pathogenicity.

**Conclusion:**

We define a novel developmental syndrome associated with biallelic hypomorphic variants in *DHRS3*; a careful assessment of individual variants is required to establish a causal link to phenotype.

## Introduction

**Figure 1:**
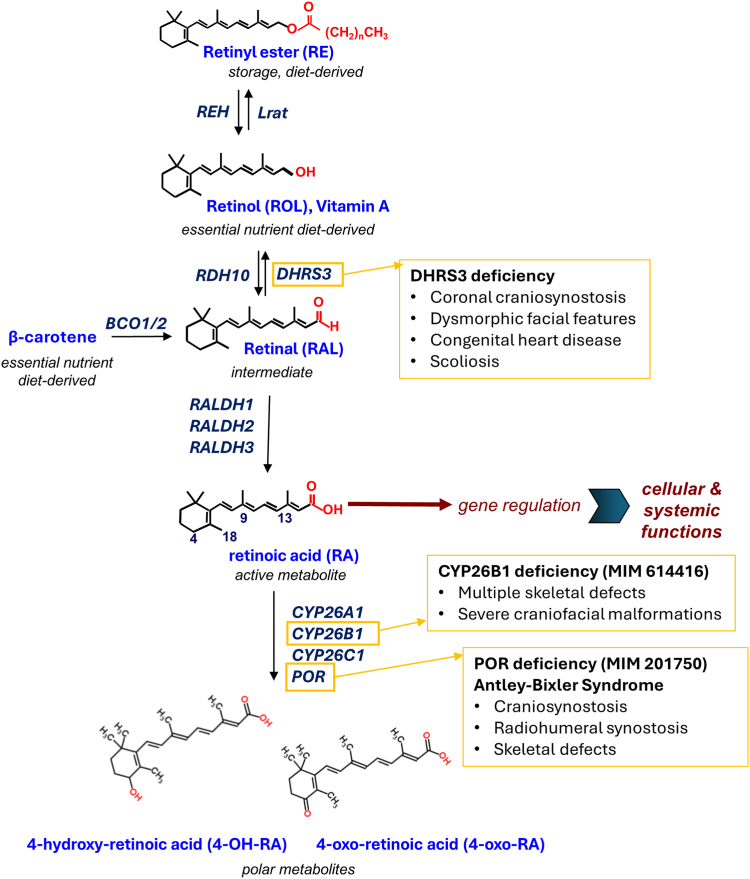
**Schematic of all-trans retinoic acid (RA) metabolism and related human congenital disorders.** RA, the active metabolite, is obligatorily derived from 2 dietary sources, all-trans-retinol (ROL) (vitamin A) and β-carotene (provitamin A) and derivatives. Oxidation of RAL, the intermediate metabolite from both of these sources, to RA occurs irreversibly, whereas oxidation of ROL to RAL is reversible, being catalyzed by related SDR16C enzymes RDH10 and DHRS3. Note that excess ROL is stored as RE. Breakdown of RA is mediated by 3 cytochrome P450 enzymes CYP26A1, CYP26B1, and CYP26C1, which all require POR as a universal electron donor. Previously identified genetic disorders of RA metabolism are indicated inside the yellow boxes (see Discussion). RAL, retinal; RE, retinyl ester.

The identification of vitamin A as an essential human nutrient and its chemical characterization in the 1930s as all-trans-retinol (ROL) provided the starting point for efforts to describe the role of retinoids in embryonic development and homeostasis.^1^ Retinoids comprise a monocyclic ring with an attached isoprenoid chain, which varies with respect to the polar end group at the terminus of the chain (Figure 1).^2^ As well as playing important roles in visual transduction and other aspects of adult homeostasis, retinoids are essential for normal embryogenesis.^3^ All-trans retinoic acid (RA) is the active metabolite of vitamin A, which typically functions in an autocrine manner. However, the lipophilic properties of this small molecule also make RA ligand well suited to act in a paracrine fashion as a morphogen.^3^^,^^4^ A corollary of this morphogenic activity is the expectation that levels of RA should be tightly regulated. Indeed, both excess and deficiency of vitamin A have serious effects on embryonic development, including an overlapping spectrum of congenital malformations involving the head, heart, and limbs.^3^^,^^5^ In particular, exposure to retinoid therapies or excessive maternal intake of vitamin A are teratogenic, being associated with an increased risk of spontaneous abortions and congenital malformations involving the craniofacial, cardiac, thymic, and central nervous systems.^6^^,^^7^ A deleterious effect on cephalic neural-crest cell activity in the early embryonic period contributes to the underlying mechanism.^8^, ^9^, ^10^

Signaling by RA is mediated by binding to a RA receptor (RAR, RARα, β, or γ) dimerized with a retinoid X receptor, leading to the altered transcription of hundreds of target genes harboring RA response elements in their enhancer regions.^11^ The mechanisms that regulate embryonic RA levels—and hence signaling—within tight limits are complex, including important roles for blood transporters (ROL-binding proteins), cell-surface receptors, cellular ROL- and RA-binding proteins, intracellular storage by esterification, and degradative pathways.^12^ A simplified summary of the core biochemical steps in synthesis and disposal of RA is illustrated in Figure 1. Dietary precursors of RA are derived from 2 major sources, preformed vitamin A such as ROL (mainly animal sources) and provitamin A carotenoids, such as β-carotene (vegetable sources). Conversion of the alcohol derivative ROL to RA involves 2 successive oxidation steps, via the aldehyde all-trans-retinaldehyde (RAL; Figure 1). Although the second step (catalyzed by 3 RAL dehydrogenases 1, 2, and 3) is rapid and irreversible, oxidation of ROL to RAL is rate limiting and reversible and therefore represents the major regulatory point during synthesis. The forward reaction is catalyzed by several ROL dehydrogenases, including, most critically for embryogenesis, RDH10,^13^, ^14^, ^15^ whereas dehydrogenase/reductase 3 (DHRS3, also named SDR16C1; encoded by *DHRS3*, HGNC:17693) is the major embryonic enzyme catalyzing the opposite reaction (RAL to ROL).^12^ RDH10 and DHRS3 function together as a molecular complex inserted within the membrane of the endoplasmic reticulum.^16^ Evolutionarily, they are closely related enzymes belonging to the SDR16C subfamily within the extensive short-chain dehydrogenase/reductase superfamily, but nevertheless, both have ancient origins with corresponding homologs identifiable in all of the vertebrates that were examined.^17^^,^^18^

Illustrating the critical importance of DHRS3 for regulation of RA, ablation of the orthologous gene in mice by deletion of exon 1 (*Dhrs3*^*−/−*^) increased the level of embryonic RA by approximately 40% and decreased ROL and retinyl esters (REs) by approximately 60% and 55%, respectively.^19^ Furthermore, homozygous *Dhrs3*^*−/−*^ embryos die during gestation (between embryonic day 14.5 and at birth) and present with defects in cardiac outflow tract formation, atrial and ventricular septation, skeletal development, and palatogenesis.^19^ Loss-of-function studies have also confirmed the function of DHRS3 homologs from zebrafish and Xenopus in controlling RA levels.^20^^,^^21^ Although it is therefore known that DHRS3 is essential to maintain correct levels of RA for normal embryonic development across a range of vertebrate species, the effects of reduced functional output of the orthologous human *DHRS3* gene have not been described.

Here, we identified 8 sibships comprising 12 individuals segregating 5 different homozygous variants of *DHRS3*. By comparative analysis of associated phenotypes, plasma measurements of RA, and other retinoids, together with an in vitro assessment of enzyme activity, we determined that 5 of the individuals (harboring 2 different variants) display features consistent with a novel RA embryopathy. Shared phenotypes include craniosynostosis, scoliosis, and structural heart defects, features congruent with further phenotyping of the corresponding mouse *Dhrs3*^*−/−*^model.

## Materials and Methods

### Clinical studies

Family 1 was recruited into the Genetic Basis of Craniofacial Malformations study according to the clinical protocol approved by London Riverside Research Ethics Committee (reference 09/H0706/20). Individuals homozygous for DHRS3 missense variants were subsequently identified by personal communication or after the submission of a *DHRS3* gene request to GeneMatcher^22^ or by interrogation of the Genomics England 100,000 Genomes Project^23^ and gnomAD databases.^24^ Analysis of family 2 was approved by the ethics committee at Philadelphia University-Jordan. Families 3 to 5 were recruited and analyzed as part of clinical diagnostic studies. Written consent to undertake the work and separate written consent for the publication of clinical photographs was obtained from patients or their parents in all cases. The 100,000 Genomes Project was approved by East of England-Cambridge South Research Ethics Committee.

### Genome analysis

Exome sequencing (ES) was undertaken on samples from 6 individuals in families 1 to 5; in addition, genome sequencing (GS) was performed on a sample from individual III-1 in family 1. Further details on exome capture, sequencing platform, mapping, variant calling, annotation, and filtering are provided in Supplemental Table 1. The sequence findings in 2 of the families were published previously in brief outline (family 1 as part of the OxClinWGS cohort [ref. 007Cra001]^25^ and family 2 [ref. family 9]).^26^ In individual III-1 in family 1, a high-resolution single-nucleotide polymorphism (SNP) array was undertaken using the Illumina Infinium Omini2.5 Exome v1.2 chip (total number of markers 2,612,357) and analyzed using Nexus Copy Number. The 3 individuals homozygous for p.(Gly115Asp) were identified by interrogating the gnomAD v4^24^ and 100,000 Genomes Project^27^ data sets.

### Targeted analysis of *DHRS3*

Resequencing of *DHRS3* was performed in a cohort of 355 patients with craniosynostosis for whom previous genetic testing had yielded normal results. Primer pairs were designed to include the entire open reading frame of *DHRS3*, and an additional primer pair was included to identify the deletion present in family 1. Further details on the method of resequencing and primers used are provided in the Supplemental Methods and Supplemental Table 2, respectively. Dideoxy sequencing was performed using standard protocols to identify the breakpoint in family 1.

### Quantitative real-time reverse transcriptase polymerase chain reaction

Whole blood samples were collected into PAXgene Blood RNA tubes and the PAXgene Blood RNA Kit (PreAnalytiX) was used to extract the RNA. RNA was extracted from lymphoblastoid cell lines using Trizol reagent (Invitrogen) and the RNeasy kit (Qiagen). Complementary DNA synthesis was performed with the RevertAid First Strand Synthesis kit (Thermo Fisher Scientific) using either oligo (dT) primers or random hexamer primers, according to the manufacturer’s instructions. Quantitative real-time polymerase chain reaction (PCR) using primers to *DHRS3* was performed using the KAPA SYBR FAST qPCR kit (Kapa Biosystems) according to the manufacturer’s instructions on a LightCycler480 II, with *ACTB* (HGNC:132) and *HPRT1* (HGNC:5157) providing endogenous controls (primer pairs in Supplemental Table 2). The results were analyzed by the delta-delta Ct method.^28^

### Quantification of retinoid metabolites in patient samples

Whole blood was collected in ethylenediaminetetraacetic acid tubes and plasma separated by centrifugation at 4 °C, under conditions designed to minimize exposure to ultraviolet light. Plasma was stored in 0.2 ml aliquots at −80 °C until analysis. Extraction of retinoids was performed using 100 to 200 μL of plasma in each replicate with multiple aliquots independently extracted and analyzed from each patient. Extraction was performed under yellow lights using a 2-step liquid-liquid extraction, with 4,4-dimethyl-RA as an internal standard for RA and retinyl acetate as an internal standard for ROL and total RE.^29^, ^30^, ^31^, ^32^, ^33^ All authentic retinoid standards were obtained from Sigma-Aldrich except 4,4-dimethyl-RA, which was obtained from Toronto Research Chemicals. Concentrations of RE and ROL were determined using high-performance liquid chromatography with ultraviolet spectroscopy with an ACQUITY H-Class Ultra-HPLC equipped with a photodiode array detector (Waters).^30^^,^^32^^,^^33^ Concentrations of RA were determined using liquid chromatography-multistage-tandem mass spectrometry using atmospheric pressure chemical ionization in positive-ion mode with a 6500+ QTRAP hybrid tandem quadruple mass spectrometer (AB Sciex).^34^ Plasma retinoids are expressed as moles per milliliter of plasma and shown as mean ± SD of measurements of individual aliquots from a given patient. Retinoid levels in patients were compared with levels in unaffected relatives and, in some cases, unrelated controls from the same geographic area, using a one-way analysis of variance with Tukey’s correction for multiple testing in GraphPad Prism V6.07. Notional reference interval ranges for each family as a subpopulation that define excess and deficiency were calculated using the % critical difference in Söderlund et al^35^ and the average wild-type (WT) levels in each family subpopulation. If WT were not available in a family subpopulation, average levels in heterozygotes were used as the control to determine the notional reference interval of the family subpopulation (Supplemental Methods).

### Modeling of DHRS3 structure

We performed in silico computational analysis of each *DHRS3* variant using Dynamut2 (biosig.lab.uq.edu.au/dynamut2/) and FoldX (foldxsuite.crg.eu) to calculate any change in free energy relative to WT using the ΔΔG method. Images of predicted *DHRS3* variant structures, including cofactor nicotinamide adenine dinucleotide phosphate, were generated using AlphaFold 3^36^ in PyMOL version 3.0.0 (Schrödinger, LLC) and Missense3D (http://missense3d.bc.ic.ac.uk/).^37^

### Cellular studies of dehydrogenase/reductase 3 activity

To determine the impact of the DHRS3 variants on enzymatic activity, HEK293 cells were stably transfected with 15 μg WT *DHRS3*, *DHRS3*^V110I^, *DHRS3*^G115D^, *DHRS3*^V171M^, and *DHRS3*^E244Q^ generated through gene synthesis and cloned in the pIRES2-green fluorescent protein (GFP) expression plasmid (Bio Basic Inc); all constructs were verified by dideoxy sequencing. Cells stably expressing WT or variant DHRS3 were cultured in Corning minimum essential media (10-009-CV, VWR) with fetal bovine serum (10437028, Gibco) and G418 sulfate (Sigma-Aldrich), from which 0.2 × 10^6^ cells were seeded in 6-well plates for each cell line. The cell media was then replaced with serum-free media to remove any serum-derived retinoids, and the cells were dosed with 1-μM RAL or vehicle (ethanol) and incubated at 37 °C for 3 hours. After incubation, medium was aspirated from the cells and each 6-well plate was wrapped in aluminum foil and stored at −80 °C until required. Upon thawing, cells were resuspended in 0.8 ml 0.9% NaCl (normal saline), and retinoids (RE, ROL, and RA) were quantified in cell pellets using high-performance liquid chromatography with ultraviolet spectroscopy for ROL and total RE and liquid chromatography-multistage-tandem mass spectrometry for RA, as described above.^30^^,^^32^, ^33^, ^34^ To control for variance in DHRS3 expression between constructs, images of each well of HEK293 cells were taken using an Invitrogen EVOS FL microscope using the GFP filter at 10x magnification and imported as TIFF files into ImageJ 1.54f (http://imagej.org). A color histogram was applied to calculate the average amount of green fluorescence, gMean, across each image. Retinoid levels were normalized according to cellular GFP fluorescence, calculated from the gMean normalized to the pixel count (255 pixels), which is proportional to the expression of DHRS3. Activity data are expressed as mean ± SD with *n* representing retinoid values for individual cell samples (independent wells). Statistical significance was assessed via 2-tailed, unpaired *t* test of each variant compared with WT (*n* = 5-6 independent biological replicates), using GraphPad Prism V6.07.

### Mouse husbandry and skeletal staining

*Dhrs3*^+/−^ mice were maintained and genotyped as previously described.^19^ All animal experiments were conducted in accordance with the approved protocols of the University of Kansas Institutional Animal Care and Use Committee. Briefly, mice were maintained on a defined diet containing 4 IU/g of preformed vitamin A (as retinyl palmitate), which represents an adequate yet moderate amount of vitamin A compared with the copious amount of preformed vitamin A in chow (15 IU/g-30 IU/g, depending on the source).^38^ For timed matings, active *Dhrs3*^+/−^ male breeders and *Dhrs3*^+/−^ females in estrus were paired overnight, and the day a vaginal plug was observed was designated as embryonic day (E) 0.5. *Dhrs3*^−/−^ mice die during late gestation; therefore, pregnant dams were euthanized, and embryos were collected at E14.5-17.5. For genotypic analysis, genomic DNA was prepared from yolk sacs, whole embryos, or mouse tails as previously described.^19^ Skeletal preparation of mouse embryos is described in the Supplemental Methods.

## Results

### Homozygous deletion including promoter and 5′ untranslated region of *DHRS3*

**Figure 2:**
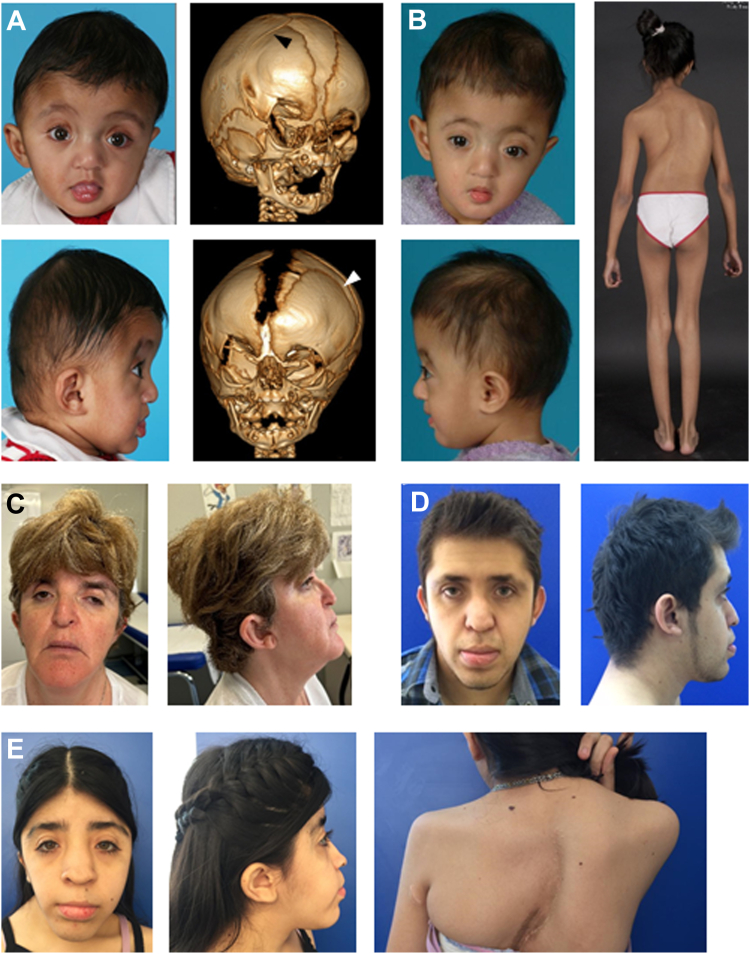
**Clinical features of patients harboring homozygous *DHRS3* variants.** A. III-4 and (B) III-1 from family 1, with promoter/5′-UTR deletion. In (A), facial appearance at age 6 months, 3-dimensional computed tomography scan at 1 month. Note fused right coronal suture (black arrow, top image) and widely patent left coronal suture (white arrow, bottom image). B. Facial appearance at age 6 months, posterior view showing scoliosis at 10 years. C-E. Individuals with p.(Val171Met) substitution. C. Facial appearance of III-2 from family 3 aged 52 years. D. Facial appearance of II-1 from family 4 aged 22 years. E. Facial appearance of II-2 from family 4 aged 16 years and (right) view of upper back showing scoliosis. UTR, untranslated region.

Initially we sought a suspected underlying genetic basis for the features present in female and male siblings from family 1 (III-1 and III-4, respectively), born to first-cousin parents from Pakistan (for pedigrees of each family; Supplemental Figure 1). The siblings both presented with a combination of unilateral or bilateral coronal craniosynostosis, atrial septal defect, and thoracolumbar scoliosis, suggesting a shared autosomal recessive basis to their disorder. The clinical features are summarized in Table 1, clinical photographs in Figures 2A and B and Supplemental Case Reports are provided in the Supplemental Material. After diagnostic testing of III-4 by chromosomal microarray and targeted variant screening of genes commonly implicated in craniosynostosis had failed to identify a diagnosis, we combined the ES of the sample from III-4, GS of III-1, and high-resolution SNP genotyping of III-1. We analyzed the data based on the hypothesis that both affected individuals, but not their unaffected sibling III-2, would share homozygosity for the same pathogenic variant.

**Table 1:** Clinical assessment of individuals homozygous for variants in *DHRS3*^a^

Family	Country of Origin	DNA Variant^b^	Protein Variant^b^	Individual	Sex	Craniofacial			Cardiac	Thoracolumbar Scoliosis	Brain Imaging	Developmental Delay/Intellectual Disability	Additional Features
						Craniosynostosis	Midface Hypoplasia	Other					
1	Pakistan	g.12617576_12621501del	NA	III-1	F	BC, ?LLa	+	Hypertelorism, broad nasal bridge, bulbous nasal tip	Atrial septal defect	+	Normal	Normal	Psoriasis, vesicular milia-like lesions on either side of the nose, long fingers
				III-4	M	RUC	−	Hypertelorism, broad nasal bridge, bulbous nasal tip, short palpebral fissures	Atrial septal defect, patent foramen ovale	+	Ventricular dilatation, Cerebellar vermis hypoplasia	Moderate	Bilateral cryptorchidism, hypospadias, long fingers, persistent feeding difficulties requiring nasogastric tube feeding, bilateral conductive hearing impairment, disordered breathing during sleep
2	Jordan	c.328G>A	p.(Val110Ile)	III-2	F	−	−	−	−	−	na	Moderate	−
				III-3	F	−	−	−	−	−	na	Moderate	−
				III-4	M	−	−	−	−	−	na	Moderate	−
3	Spain	c.511G>A	p.(Val171Met)	II-2	F	UC	+	Hypertelorism, proptosis, mild microcephaly	Aortic pseudo-coarctation/root dilatation, absent pericardium	+	na	Mild	Right inguinal hernia, mild conductive hearing impairment
4	Mexico	c.511G>A	p.(Val171Met)	II-1	M	BC	+	Mild proptosis, unilateral microtia	Atrial septal defect	+	na	Mild	Thenar and hypothenar hypoplasia, carpal fusions, moderate sensorineural hearing loss
				II-2	F	BC	+	Mild proptosis	−	+	na	Mild	Congenital anal fistula, carpal and tarsal fusions, moderate sensorineural hearing loss
5	Syria	c.730G>C	p.(Glu244Gln)	III-1	M	−	−	Macrocephaly, severe hypertelorism, R cleft lip, L cleft palate	−	+	Agenesis of corpus callosum, occipital meningocele, schizencephaly, absent cerebellar vermis, dysplastic cerebellar hemispheres	Severe	Short stature, epilepsy, severe visual impairment with divergent strabismus and nystagmus

*+*, present; *−*, absent; *BC*, bicoronal; *F*, female; *L*, left; *La*, unilambdoid; *M*, male; *na*, not available; *NA*, not applicable; *R*, right; *UC*, unicoronal.

a Omits 3 individuals homozygous for the c.344G>A [p.(Gly115Asp)] variant, who were not assessed clinically as part of this study (see main text).

b All tabulated variants were present in homozygous state; for brevity, formal biallelic notation and cDNA reference (NM_004753.7) are not included. For full nomenclature details of each variant, see Table 2.

**Figure 3:**
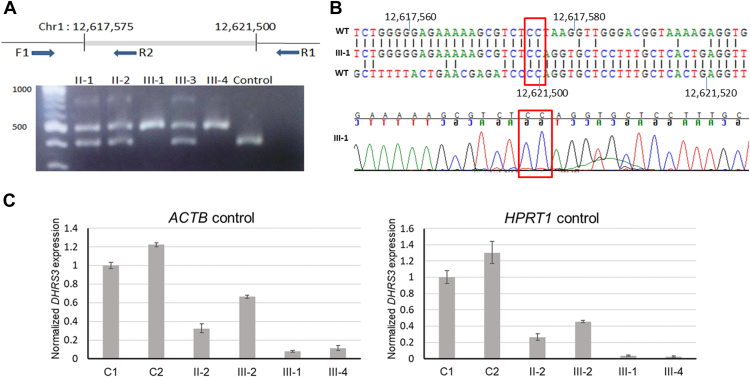
**Homozygous deletion of promoter and part of 5′-UTR in family 1 abrogates *DHRS3* expression.** A. Upper panel, schematic of the deletion breakpoints showing positions of primers used for polymerase chain reaction (PCR) analysis. Lower panel, genotyping of family 1 members using PCR-containing primers F1, R1, and R2; predicted sizes of F1-R1 and F1-R2 products are 506 bp and 314 bp, respectively. Faint product at top of gel represents a heteroduplex product in heterozygous samples. Note that the unaffected sibling III-2 is heterozygous for the deleted allele. B. Upper panel, alignment of sequences at proximal breakpoint (top) and distal breakpoint (bottom), sandwiching the observed breakpoint sequence. Nucleotide identities are connected by solid lines. Lower panel, dideoxy-sequence chromatogram at the breakpoint. Red boxes outline the shared 2-nucleotide identity at the proximal and distal breakpoints. C. Real-time reverse transcriptase PCR analysis of *DHRS3* expression based on complementary DNA derived from whole blood, in 2 control samples (C1 and C2), 2 heterozygotes for the deletion (II-2 and III-2), and the 2 affected children (III-1 and III-4). Results are plotted as mean ± SD of 3 biological replicates, normalized to either *ACTB* (left) or *HPRT1* (right) expression and calculated relative to the mean value of the C1 sample (=1). UTR, untranslated region.

Comparison of ES data from III-4 with GS data from III-1 revealed 13 shared regions of homozygosity > 2 Mb, totaling approximately 106 Mb in extent (data not shown). After filtering for homozygous predicted protein-altering variants with allele frequency < 0.005 and supported by >5 reads in both ES and GS data sets, 4 variants were shared between the affected siblings. Investigation of the unaffected sibling III-2 revealed that all 4 variants were also identically homozygous in this individual, eliminating a causal role based on our hypothesized disease mechanism (Supplemental Table 3). We then performed high-resolution SNP genotyping of III-1, seeking copy number variants that had not been identified in the previous microarray or sequencing data. Of the 27 apparently homozygous deletions initially called based on signal dropout, only 3 were rare, verified by the examination of corresponding GS data and predicted to be present in III-4 based on shared regions of homozygosity (Supplemental Table 4). Two of these were located >10 kb from any exon; therefore, we focused on the remaining deletion, which included part of exon 1 of *DHRS3* (Supplemental Figures 2A-C). By targeted PCR and dideoxy sequencing, we confirmed a deletion of 3926 bp extending into exon 1 of *DHRS3* (NM_004753.7; ENST00000616661.5) and demonstrated segregation in the pedigree consistent with a causal role (Figure 3A). The deletion was annotated as NC_000001.11:g.12617576_12621501del (GRCh38), with 2 bp ambiguity at the breakpoint consistent with microhomology-mediated end-joining mechanism of origin (Figure 3B). No similar deletion is annotated in gnomAD SVs v4.0.^39^

**Figure 4:**
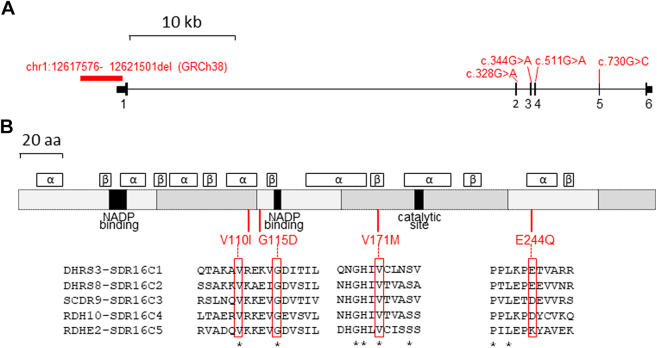
**Structure of *DHRS3* and encoded protein and location of homozygous variants.** A. Scale diagram of gene showing exons numbered 1 to 6. Taller vertical lines denote the coding regions of exons, flanked by 5′- and 3′-UTRs at each end. The positions of the 3926-bp deletion and 4 nucleotide substitutions are indicated in red. B. Cartoon of protein. Alternating gray blocks of different shades indicate regions encoded by sequential exons. Black segments show regions involved in enzymatic functions. Above the cartoon are shown regions predicted by AlphaFold to adopt either –α-helix or β-sheet structure with a per-residue confidence score >90. Below the cartoon, the locations of the 4 amino acid substitutions are shown with local alignments of the 5 members of the human SDR16C family (red rectangles).^40^ Asterisks denote invariant amino acids. Note that DHRS8 and SCDR9 are synonymous with HSD17B11 and HSD17B13, respectively. aa, amino acid; UTR, untranslated region.

The deletion abolishes conserved sequences within the promoter together with the first 635 nucleotides of the 5′-untranslated region **(**UTR) of *DHRS3* (Figure 4A^40^, Supplemental Figure 2D), a gene comprising 6 exons. We anticipated that expression of *DHRS3* would likely be severely abrogated, but any residual transcript would encode a protein of normal sequence and function. To investigate *DHRS3* expression, we used real-time reverse transcriptase-PCR to quantify levels of transcript in whole blood cell RNA isolated from the 2 affected individuals III-1 and III-4. Using 2 different reference complementary DNA controls (*ACTB* and *HPRT1*) for normalization, *DHRS3* transcripts were expressed on average at 0.022-to-0.10-fold levels in whole blood, compared with 2 control samples; heterozygotes showed intermediate levels of expression (Figure 3C). Similar data were obtained for lymphoblastoid cell lines from the 2 patients (0.013-0.052-fold level of *DHRS3* expression compared with 2 controls; Supplemental Figure 3). These results are consistent with severe (90%-98%) abrogation of DHRS3 enzyme activity in these patients. To corroborate this finding, we sought further individuals with phenotypes potentially related to DHRS3 deficiency and undertook in vivo and in vitro experiments to investigate the consequences for RA metabolism.

### Identification of homozygous missense variants in *DHRS3*

Given that both affected siblings in family 1 presented with craniosynostosis, we first undertook resequencing and targeted PCR analysis of *DHRS3* in 355 unrelated individuals with craniosynostosis of undetermined cause, including 32 of South Asian ancestry. However, this did not reveal further examples either of the same 5′-UTR deletion as found in family 1, nor of homozygosity or compound heterozygosity for any rare coding variants in *DHRS3* (data not shown).

As alternative approaches, we used phenotype-independent methods to find additional individuals with relevant *DHRS3* variants (see Materials and Methods). These approaches led to the identification of 7 additional sibships comprising 1 or more individuals harboring rare (maximum allele frequency in any gnomAD population 0.00145) homozygous missense variants of *DHRS3* (Table 2, Supplemental Figure 4). These comprised (1) a previously reported^26^ Jordanian family (family 2) in which 3 siblings with learning disability were homozygous for NC_000001.11:g.12580534C>T encoding p.(Val110Ile), (2) 2 unrelated families (families 3 and 4) homozygous for NC_000001.11:g.12578905C>T encoding p.(Val171Met), comprising 1 or 2 affected individuals each with craniosynostosis similar to family 1, and (3) a single individual homozygous for NC_000001.11:g.12572822C>G encoding p.(Glu244Gln) with a severe craniofacial and neurodevelopmental phenotype (family 5). Pedigrees for each of these families are shown in Supplemental Figure 1 and additional rare homozygous variants identified in the proband from family 5 are listed in Supplemental Table 5. We also included (but did not assess clinically) 3 apparently unrelated individuals homozygous for NC_000001.11:g.12579408C>T encoding p.(Gly115Asp), 2 of whom were classified as phenotypically normal/unaffected; these individuals were identified in either the gnomAD or 100,000 Genomes Project databases. Although not directly implicated in catalysis, 3 of the 4 amino acid residues subject to substitutions (p.Val110, p.Gly115, and p.Val171) are completely conserved in all 5 members of the human SDR16 family (Figure 4B^40^), implying important roles in the integrity of protein function.

**Table 2:** Homozygous variants of *DHRS3* assessed in this work

Variant^a^	Consequence	dbSNP ID	gnomAD v4.1.0 Maximum MAF	CADD Score (GRCh38)	REVEL Score (GRCh38)	AlphaMissense Score	Ascertainment	Country/Region of Ancestry	Homozygous Individuals	Phenotypic Information (Table 1)	RA Measurements	Functional Analysis (RNA or Expression Constructs)	Structural Modeling	ACMG Criteria	Pathogenicity (See Discussion)
g.12617576_ 12621501del	3926 bp partial deletion promoter and 5′-UTR	NA	Absent	NA	NA	NA	Index family	Pakistan	Family 1: 2 affected siblings	Y	Y	Y	na	PS1, PS3, PP4	Pathogenic
g.12580534C>T; c.328G>A	p.(Val110Ile)	rs200750969	0.0015 Middle Eastern	24.5	0.38	0.19 likely benign	GeneMatcher	Jordan	Family 2: 3 affected siblings	Y	Y	Y	Y	BS3	Likely benign
g.12579408C>T; c.344G>A	p.(Gly115Asp)	rs749951435	0.00015 South Asian	27.0	0.86	0.86 likely pathogenic	gnomAD v3.1.1	South Asian	1 singleton	control/biobank sample	N	Y	Y	PP3, BS2	Likely benign
							100kGP	South Asian	1 father	unaffected				PP3, BS2	
							100kGP	South Asian	1 of 2 affected siblings	N (potentially coincidental observation)				PP3, BS4	
g.12578905C>T; c.511G>A	p.(Val171Met)	rs1488627630	0.00013 Finnish	27.4	0.78	0.96 likely pathogenic	GeneMatcher	Spain	Family 3: 1 affected individual	Y	Y	Y	Y	PS1, PS3, PP3, PP4	Pathogenic
							Personal communication	Mexico	Family 4: 2 affected siblings	Y	N			PS3, PP3, PP4	
g.12572822C>G; c.730G>C	p.(Glu244Gln)	rs1389365121	0.0005 Middle Eastern	22.8	0.59	0.18 likely benign	GeneMatcher	Syria	Family 5: 1 affected individual	Y	Y	Y	Y	PS3	VUS

*100kGP*, 100,000 Genomes Project; *ACMG*, American College of Medical Genetics; *cDNA*, complementary DNA; *dbSNP*, single nucleotide polymorphism database; *gDNA*, genomic DNA; *ID*, identification; *MAF*, minor allele frequency; *NA*, not applicable; *N*, no; *RA*, retinoic acid; *UTR*, untranslated region; *VUS*, variant of uncertain significance; *Y*, yes.

a All tabulated variants were present in homozygous state; for brevity, formal biallelic notation and respective gDNA and cDNA references (NC_000001.11 and NM_004753.7) are not included in this column. The cDNA notation for the deletion in family 1 is NC_000001.11(NM_004753.7):c.-4151_-226del. Genomic coordinates are derived from the GRCh38 reference.

To explore further the potential pathogenicity of these missense variants, we combined bioinformatic assessment (Table 2), clinical phenotyping (Table 1 and Supplemental Case Reports), and biochemical analysis (next section). For the in silico pathogenicity assessment, we considered 2 well-established severity scores, CADD^41^ and REVEL,^42^ together with the more recently published AlphaMissense classifier.^43^ All 3 measures concur that the consequences of 2 substitutions (p.Gly115Asp and p.Val171Met) are likely more severe than the other 2 (p.Val110Ile and p.Glu244Gln); AlphaMissense classified the first 2 as likely pathogenic and the second 2 as likely benign (Table 2). FoldX predicted all 4 missense variants to be destabilizing, and ΔΔG values (kcal/mol) calculated by Dynamut2 were −0.98, −0.9, −0.7, and −0.62 for p.Gly115Asp, p.Val171Met, p.Glu244Gln, and p.Val110Ile, respectively. These conclusions are consistent with molecular modeling of the predicted 3-dimensional structure of DHRS3 (Supplemental Figure 5).

The phenotypes associated with the 4 homozygous missense variants are notably divergent (Table 1). The 3 individuals in families 3 and 4 homozygous for p.(Val171Met) have strikingly similar features to the siblings in family 1, including unilateral or bilateral coronal craniosynostosis (3 of 3 individuals), a similar facial appearance reminiscent of Saethre-Chotzen syndrome (Figures 2C-E), scoliosis (3 of 3), and congenital heart disease (2 of 3). In contrast, 2 of 3 individuals homozygous for p.(Gly115Asp) (classified computationally as the other severe missense variant) had normal phenotypes. The third individual, with a severe neurodevelopmental phenotype, had an affected sibling with overlapping clinical features who was heterozygous for the *DHRS3* variant, raising the possibility that the homozygous p.(Gly115Asp) could be a coincidental finding. The phenotypes in families 2 and 5 were different from families 1, 3, and 4 (summarized in Table 1; more details in the Supplemental Case Reports). Given that the affected individuals were the offspring of consanguineous unions, the possibility of a separate pathology for their disorders could not be fully excluded. To assist in disentangling these genotype-phenotype relationships, we measured RA metabolites in blood plasma samples from selected individuals and undertook in vitro studies of the enzyme activity, as summarized in Table 2.

### Retinoid metabolites measured in individuals homozygous for *DHRS3* variants

**Figure 5:**
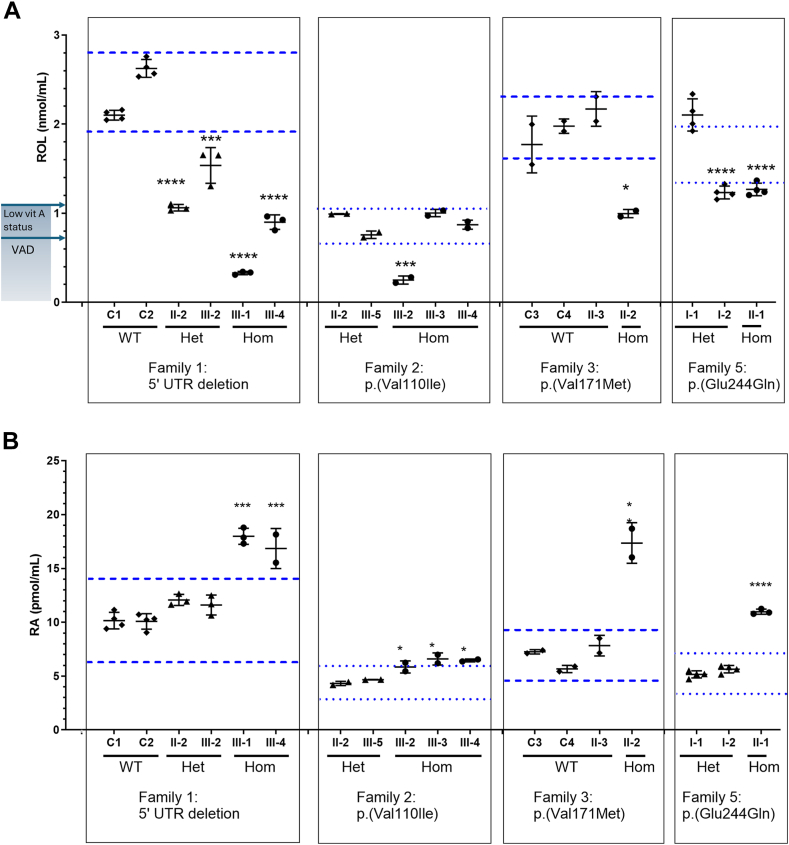
**Measurement of all-trans-retinol (ROL) and all-trans retinoic acid (RA) in plasma of individuals from families 1, 2, 3, and 5.** A. ROL (nmol/mL) levels for each set of family samples were determined by high-performance liquid chromatography with UV spectroscopy. B. RA measurements (pmol/mL) determined by liquid chromatography-multistage-tandem mass spectrometry. The *x*-axis labels indicate the identity of the individual analyzed (see pedigrees in Supplemental Figure 1) and, below, whether they are wild-type (WT), heterozygous (Het), or homozygous (Hom) for the corresponding *DHRS3* variant. Additional unrelated WT controls C1-C4 were included in the analyses of families 1 or 3. Limits for excess and deficiency (parallel blue lines) were calculated for each family as a subpopulation using the average WT (dashed lines) or average Het (dotted lines) levels and the critical difference from Söderlund et al.^35^ In part A, plasma retinol levels that define vitamin A deficiency (VAD, 0.7 nmol/mL) and low vitamin A status (0.7-1.05 nmol/mL) are notated on the *y*-axis.^44^ Significance of differences of Hom cases within individual families, compared with unaffected individuals, were determined by one-way ANOVA with Tukey’s correction for multiple comparisons. ∗*P* = .05-.01, ∗∗∗*P* < .001, ∗∗∗∗*P* < .0001. A detailed commentary on these results is available in Supplemental Results: Plasma Retinoid Profile. ANOVA, analysis of variance; UTR, untranslated region.

Plasma retinoids, including ROL and RA, were quantified in family 1 (5′-UTR deletion), family 2 (p.(Val110Ile)), family 3 (p.(Val171Met)), and family 5 (p.(Glu244Gln)), compared with siblings, parents, and simultaneously sourced controls. ROL (Figure 5A^35^^,^^44^) is vitamin A, and the end-product of the DHRS3 enzymatic reduction of intermediate RAL; RA (Figure 5B^35^^,^^44^) is the main active metabolite of vitamin A that regulates gene transcription and is teratogenic. Because DHRS3 catalyzes the reduction of RAL to ROL, thereby limiting RA production, loss of DHRS3 function would lead to the unfettered, irreversible oxidation of RAL into RA (Figure 1). For completeness, other endogenous retinoids in plasma (endogenous RA isomers and RE) were also quantified (Supplemental Figures 6-9, Supplemental Table 6). Taking account of the fact that baseline variation in retinoid nutritional status may affect RA, we first interrogated ROL levels. Each family was stratified into a unique subpopulation and homozygous individuals compared with similarly sourced controls in which thresholds for excess or deficiency were defined according to the reported critical difference at the 95% confidence level (Supplemental Table 7).^35^^,^^45^

The results of ROL measurements are shown in Figure 5A.^35^^,^^44^ Homozygous individuals in families 1 (5′-UTR deletion) and 3 [p.(Val171Met)] had significantly lower plasma ROL than simultaneously obtained WT controls; in family 1, ROL levels were also lower in heterozygous individuals than WT controls. Within each family, ROL levels in homozygotes were lower than, or similar to, those in heterozygous individuals. Notably, in 2 homozygotes (III-1 in family 1 and III-2 in family 2 [p.(Val110Ile)]), the ROL levels were below the threshold for diagnosis of vitamin A deficiency. These data showing reduced plasma ROL are consistent with diminished catalytic activity of DHRS3.

An important role of DHRS3 reduction of RAL to ROL is to limit RA production (Figure 1). As such, plasma RA was quantified to assess the DHRS3 variants (Figure 5B^35^^,^^44^). In all 4 families, plasma levels of RA in the homozygous individuals were significantly higher than for heterozygous individuals from the same family or simultaneously obtained unaffected control samples. Control plasma RA levels differed substantially between families, consistent with the need for stratification of each family as a unique subpopulation driven by nutritional environment. Of note, in family 1 (5′-UTR deletion), homozygotes III-1 and III-4 had significantly greater plasma RA than WT and exceeded the statistical threshold for excess for the subpopulation, indicating pathologic RA excess. In family 2 (p.(Val110Ile)), which is from the most nutritionally limited environment, there was the lowest magnitude increase in plasma RA in the 3 homozygous individuals, but nevertheless, these were significantly increased compared with 2 heterozygotes. In family 3 (p.(Val171Met)), homozygote II-2 had the largest increase in RA compared with WT and exceeded the upper-limit-defining excess for the subpopulation, indicating the pathologic excess of plasma RA. In family 5 (p.(Glu244Gln)), RA in the homozygote II-1 was significantly greater than in either heterozygote, and exceeded the upper subpopulation statistical threshold for excess.

In summary, these data indicate that all 4 variants analyzed are functionally significant and diminish the catalytic activity of DHRS3, consistent with computational modeling. Of note, the magnitude of the observed increase in plasma RA differed substantially between families. In families 1 (5′-UTR deletion) and 3 (p.(Val171Met)), the average levels of plasma RA in homozygotes were 72% and 151% greater, respectively, than unaffected WT individuals in their respective family subpopulations. In family 2 (p.(Val110Ile)), RA levels in homozygotes were 40% increased over heterozygotes, a lesser increase compared with families 1 and 3. In family 5 (p.(Glu244Gln)), the homozygous individual had 103% increase over the average heterozygous RA level. The plasma RA magnitude of change in homozygotes compared with unaffected WT or heterozygotes is consistent with the severity of the phenotype. Further description and interpretation of the plasma retinoid profiles are provided in the Supplemental Results.

### Measurement of dehydrogenase/reductase 3 activity

**Figure 6:**
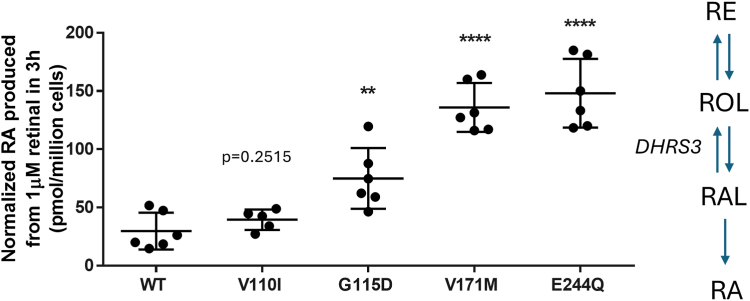
**In vitro measurement of DHRS3 activity.** Cell lines stably transfected with the variants p.Val110Ile (V110I), p.Gly115Asp (G115D), p.Val171Met (V171M), p.Glu244Gln (E244Q), or wild-type (WT) construct were treated with 1 μM RAL for 3 hours and assayed for RA production to assess the DHRS3 loss of function. Measured retinoid levels were normalized to the average green fluorescent protein (GFP) fluorescence per pixel to account for variations in DHRS3 expression (Supplemental Figure 10C). *DHRS3* variants displayed elevated RA indicating reduced DHRS3 activity. Significance of the change in activity was determined via 2-tailed, unpaired *t* test compared with WT. *n* = 5-6 independent biological replicates; ∗∗*P* < .01, ∗∗∗∗*P* < .0001. RA, retinoic acid; RAL, retinal; RE, retinyl ester; ROL, retinol.

To assess the impact of the DHRS3 variants on enzymatic activity, we stably transfected HEK293 cells with each variant and assessed the activity of DHRS3 when cells were provided with substrate, RAL. All variants except p.Val110Ile elicited a significant increase in RA (Figure 6), which was accompanied in all cases by a decrease in ROL and RE (Supplemental Figure 10), consistent with diminished function. Basal cellular retinoids mirrored the activity data (Supplemental Figure 11). The magnitude of RA dysregulation varied, with the greatest reduction in DHRS3 activity (corresponding to increase in RA) observed for the p.Val171Met and p.Glu244Gln cell lines; less-marked differences from WT were observed for the p.Val110Ile and p.Gly115Asp lines (Figure 6). These in vitro experiments are in broad agreement with the results from measurements of patient plasma and modeling predictions of instability, demonstrating that all 4 missense substitutions reduce DHRS3 function, but to different extents. Further interpretation of the in vitro cellular retinoid profile is provided in the Supplemental Results.

### Extended analysis of the murine *Dhrs3*^*−/−*^ phenotype, *Dhrs3* expression, and RA signaling in vivo

**Figure 7:**
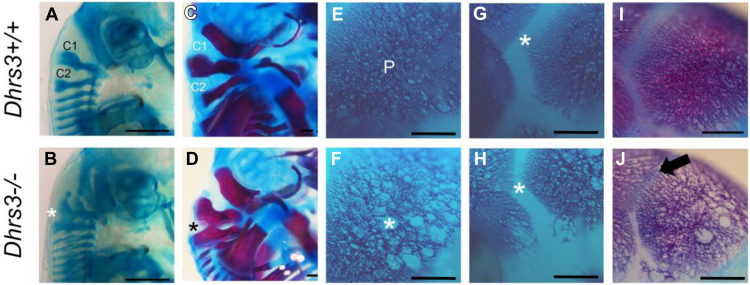
***Dhrs3*^*−/−*^ mutant mice exhibit axial and cranial skeletal anomalies.** A and B. Alcian blue staining of cartilage reveals distinct C1 and C2 vertebrae in E14.5 *Dhrs3*^*+/+*^ mouse embryos and their fusion (white asterisk) in *Dhrs3*^*−/−*^ embryos, which is consistent with upregulated RA signaling during embryogenesis. C and D. Alizarin red and Alcian blue staining of bone and cartilage, respectively, illustrates the more pronounced fusion (black asterisk) of C1 and C2 in *Dhrs3*^*−/−*^ embryos compared with *Dhrs3*^*+/+*^ embryos. E and F. Alizarin red staining of the parietal bone reveals less dense and more porous bone (white asterisk) in *Dhrs3*^*−/−*^ embryos compared with *Dhrs3*^*+/+*^ embryos. G-J. Alizarin red staining of the calvaria illustrates the narrowing of the coronal suture (white asterisk) and ectopic bone growth (black arrow) into the coronal suture in *Dhrs3*^*−/−*^ embryo compared with *Dhrs3*^*+/+*^ embryo. Scale bars, 500 μm.

To further investigate an association between DHRS3 loss of function and the phenotypes present in the affected individuals, we characterized the phenotypes of mouse embryos nullizygous for the orthologous gene (*Dhrs3*^*−/−*^). We previously reported that *Dhrs3*^*−/−*^ embryos present with defects in palatogenesis, cardiac outflow tract formation, atrial and ventricular septation, and vertebra formation and that they die during late gestation.^19^ Here, we focused on the axial and cranial skeletal phenotypes in E14.5-E17.5 late gestation embryos (Figure 7). In E14.5 *Dhrs3*^*+/+*^ embryos, the atlas and axis (C1 and C2, respectively) have distinctive shapes and are well separated from each other as evidenced by Alcian blue staining of cartilage (Figure 7A). In contrast, C1 and C2 are fused (asterisk) along their dorsal aspects in E14.5 *Dhrs3*^*−/−*^ embryos (Figure 7B). The atlas-axis fusion (asterisk) is even more pronounced after endochondral ossification in E17.5 *Dhrs3*^*−/−*^ embryos stained with Alizarin red (bone) and Alcian blue, compared with littermate *Dhrs3*^*+/+*^ embryos (Figures 7C and D). Unilateral or bilateral fusion of the first 2 cervical vertebrae, which was observed in every *Dhrs3*^*−/−*^ embryo examined (*n* = 6), is a classic feature of excessive RA signaling. E17.5 *Dhrs3*^*−/−*^ embryos also exhibited anomalies in skull bone ossification. Specifically, the parietal bones in all *Dhrs3*^*−/−*^ embryos were notably less dense and more porous, with a high frequency of circular holes (asterisk), compared with *Dhrs3*^*+/+*^ embryos (*n* = 6) (Figures 7E and F). We observed frequent unilateral (left sided) narrowing of the coronal suture (asterisk) in E17.5 *Dhrs3*^*−/−*^ embryos compared with *Dhrs3*^*+/+*^ (Figures 7G-J) and in 1 case, considerable bone growth across the coronal suture (arrow) (*n* = 1/6). Excluding the posterofrontal suture, the cranial sutures in the mouse skull remain patent throughout the life of the mouse. Therefore, although *Dhrs3*^*−/−*^ mice fail to survive to birth, their phenotypes are consistent with excess RA signaling and presage the pathogenic onset of craniosynostosis, collectively mimicking the phenotypes present in humans with variants in *DHRS3*.

To corroborate these observations, we interrogated the expression of *Dhrs3* and other key transcripts involved in RA signaling in the murine skull and assayed RA signaling output using a reporter line. A uniform manifold approximation and projection plot of *Dhrs3* expression in single-cell RNA sequencing data from E15.5 and E17.5 coronal sutures^46^ indicated prominent expression in all 4 meningeal clusters (MG1-MG4) and 1 of the 4 osteogenic clusters (OG3), with relatively lower expression in other cell types (ectocranial, ligament-like, and proliferative osteogenic clusters) (Supplemental Figure 12A). Dot plot analysis of an independently obtained calvarial and cranial suture single-cell RNA sequencing data set^47^ supports this pattern of *Dhrs3* expression (predominantly dura mater and minor osteoblast and ectocranial populations) and shows that expression of different components of vitamin A transport, RA synthesis, catabolism, and signaling are localized to different calvarial cell types (Supplemental Figure 12B). Finally, Xgal (blue) staining of a representative E18.5 *RARE-lacZ* mouse skull provides direct visualization of RA signaling in the calvaria, concentrated in the medial and ventral regions of the frontal and parietal bones on either side of the coronal sutures (Supplemental Figure 12C). Collectively, these observations further support a pathophysiological mechanism by which variants in *DHRS3* perturb retinoid metabolism and RA signaling, which results in craniosynostosis.

## Discussion

We have assessed the phenotypic and biochemical consequences of 5 different homozygous variants in *DHRS3*, which encodes an enzyme previously shown to play a critical role in RAL metabolism. Complete deficiency of *Dhrs3* in mice leads to increased embryonic tissue levels of RA accompanied by a constellation of craniofacial, cardiac, and skeletal defects consistent with excess exposure to RA during embryogenesis.^19^

Our work was initiated by the identification of 2 affected siblings in family 1 who are homozygous for a 3.9-kb deletion including part of the 5′-UTR and promoter of *DHRS3*. No alternatively spliced promoter for this gene has been described,^48^ and we documented a substantial reduction in the expression of the *DHRS3* coding region in the affected individuals, quantified in both whole blood and lymphoblastoid cell lines at between 90% and 98%. Importantly, however, residual *DHRS3* expression remained, and the resultant enzyme is expected to be intact because the open reading frame is not affected by the deletion. Hence, this represents a clear example of a severely hypomorphic (partial loss-of-function) variant.^49^ Consistent with this, measurements of RA in plasma showed marked elevation in both homozygous siblings in family 1 to levels that, if reflected in the embryo, would be expected to be toxic (Figure 5B^35^^,^^44^). Previous studies suggest that plasma RA levels can be used to evaluate teratogenic potential^50^ and that even marginal increases in the levels of RA may be detrimental.^51^

Importantly, both affected siblings in family 1 share a similar presentation of congenital anomalies, including coronal craniosynostosis, septal heart defects, and scoliosis (Figure 2, Table 1). These malformations mirror the pattern previously observed in the *Dhrs3*^−/−^ null mouse,^19^ and the proportionate increases in plasma RA in the 2 patients are comparable to the mouse observations. At e14.5, *Dhrs3*^−/−^ null embryos compared with WT had an increase in RA of 48% accompanied by a decrease in ROL of 61%. Homozygous siblings III-1 and III-4 had increases in plasma RA of 78% and 66%, respectively, which were accompanied by decreases in plasma ROL of 86% and 62%, respectively, compared with WT.

To expand upon these observations, we used multiple approaches to identify additional homozygous variants in *DHRS3* that could enrich and inform the pattern of genotype-phenotype correlation. This led to the identification of 4 different homozygous missense variants, present in a total of 10 individuals from 7 families. Interpreting the pathogenicity of individual missense variants is challenging; therefore, we combined computational evidence (Table 2), structural modeling (Supplemental Figure 5), and in vivo and in vitro biochemical analyses (Figures 5^35^^,^^44^ and 6, Supplemental Figures 6-11), alongside documentation of the phenotypes present in different individuals. Two additional potential confounders to consider were that (1) RA levels may vary according to underlying dietary availability of retinoids necessitating similarly sourced controls, and (2) most of the clinically affected *DHRS3*-homozygous individuals arose from consanguineous unions, emphasizing the need for careful search for alternative causative biallelic genetic pathology.

Dietary retinoid intake can affect baseline plasma levels of retinoids, including RA, as documented in various population studies^50^, ^51^, ^52^ and in animal models.^53^ Plasma RA has been shown to increase with consumption of food or supplements rich in provitamin A or preformed vitamin A.^50^, ^51^, ^52^^,^^54^, ^55^, ^56^, ^57^, ^58^ Relevant to potential for unique nutritional environments in the family subpopulations observed here, the nutritional adequacy of diets varies globally where high-income countries generally produce adequate amounts of dietary nutrients, whereas low-income countries often are deficient in dietary micronutrients.^59^ The families within this study for which retinoid measurements were performed were residents in United Kingdom (family 1), Jordan (family 2), Spain (family 3), and Germany (family 5). The nutrient production adequacy of these countries has been determined to be 0.8 (United Kingdom), 0.5 (Jordan), 1.3 (Spain), and 1.1 (Germany), in which values below 1 indicate a gap between regional production and requirement.^59^ It should be noted that the United Kingdom and EU countries fortify numerous foods with vitamin A, and food fortification has been shown to increase serum ROL.^60^^,^^61^ According to the plasma ROL levels, families 1, 2, 3, and 5 represent distinct nutritional subpopulations according to geographic region, with lower plasma ROL in nutritionally limited regions, eg, Jordan (family 2) (Figure 5A^35^^,^^44^). Accordingly, the baseline differences in plasma RA observed in the control groups is consistent with the differences in dietary vitamin A availability in the family subpopulations according to their nutritional environment, which justifies stratification into unique subpopulations (Figure 5B^35^^,^^44^). Importantly, our results indicate the necessity for similarly sourced controls for patient evaluation, with unaffected individuals from the same family as preferable and individuals from the same geographic-nutritional environment being acceptable.

A confident conclusion from the biochemical analyses is that all 4 missense variants (p.(Val110Ile), p.(Gly115Asp), p.(Val171Met), and p.(Glu244Gln)) diminish DHRS3 function. This is demonstrated by the in vitro analyses of RA, ROL, and RE (Figure 6, Supplemental Figures 10 and 11), which were altered in assays in the expected pattern (that is, an increase in RA and corresponding decrease in ROL and RE) for all 4 variants. These deleterious effects are consistent with structural modeling that predicted each of the variants to destabilize DHRS3 (Supplemental Figure 5). However, a deleterious effect on function does not necessarily translate to an effect on phenotype. Considering the magnitude of computationally predicted deleteriousness, plasma RA assays in patients and controls, and analysis of phenotype, we reached different conclusions about the pathogenicity of different missense variants.

The p.(Val171Met) variant, present in 3 homozygous individuals from 2 families (families 3 and 4), exhibited a pattern of findings indicating that it is pathogenic. Three scores of pathogenicity (CADD, REVEL, and AlphaMissense) uniformly assigned this as being a damaging variant (Table 2). This was supported by both in vivo and in vitro RA measurements (Figures 5^35^^,^^44^ and 6), and the phenotypes of the 3 affected individuals (with uni- or bicoronal craniosynostosis in all 3, congenital heart disease in 2 and scoliosis in all 3) are strikingly congruent to those of the siblings in family 1 (Figure 2, Table 1). Neurodevelopmental disability may be a component feature of the phenotype but tends to be mild. The Val171 residue locates in a β-strand within the Rossmann fold (Figure 4B,^40^ Supplemental Figure 5); the side chain is buried, such that substitution to the bulkier methionine is expected to be destabilizing (Supplemental Figure 5). Although we are not aware that pathogenic substitutions at the exactly equivalent position to Val171 have been described in other proteins containing Rossmann folds, pathogenic missense variants have been reported at positions located 3 to 7 residues more C-terminally in 17-β-hydroxysteroid dehydrogenase 10 (*HSD17B10*; HGNC:4800).^62^^,^^63^ Further confidence in assignment of p.(Val171Met) as pathogenic is provided by the similarity in clinical features in the 5 homozygous individuals in families 1, 3, and 4, compared with the phenotype of the mouse knockout, which also includes craniofacial, cardiac, and vertebral malformations.^19^ Here, we have extended these parallels by demonstrating that *Dhrs3*^*−/−*^ mouse embryos exhibit axial skeletal anomalies involving cervical vertebral fusion and cranial skeletal anomalies that affect parietal bone ossification, with narrower coronal sutures and the onset of craniosynostosis (Figure 7).

In contrast, the balance of evidence indicates that p.(Val110Ile) and p.(Gly115Asp) are likely benign. Available biochemical data suggest that these substitutions lead to less severe reduction in DHRS3 function (Figures 5^35^^,^^44^ and 6) and the associated phenotypes (pure intellectual disability in 3 siblings in family 2 [p.(Val110Ile)], apparently normal phenotypes in 2 of 3 individuals with p.(Gly115Asp), and inconsistent segregation of genotype with disease in the sib of the third individual) do not match the consistent phenotypic observations from families 1, 3, and 4. Of note, family 2 is from Jordan, a country where natural availability of retinoids is known to be low.^64^ All of the family members of family 2 have plasma ROL levels below typical population reference ranges for males (1.6-3.0 μmol/L) or females (1.2-2.7 μmol/L)^35^ and can be categorized as having low vitamin A status (0.7-1.05 μmol/L).^44^ In particular, homozygous III-2 has plasma ROL below the World Health Organization threshold that defines vitamin A deficiency (0.7 μmol/L).^65^ Consistent with this, levels of RA in heterozygous relatives were somewhat lower than in other families (Figure 5B^35^^,^^44^), which raises the possibility that dietary deficiency may have mitigated the occurrence of toxic RA levels in homozygous individuals. In support of this idea, not only embryonic lethality, but also vascular defects present in *Dhrs3*^−/−^ mutant embryos can be prevented by reducing the vitamin A content of the mother’s diet, which indicates that the phenotype elicited by *Dhrs3*-ablation is highly responsive to the level of ROL precursor available for RA synthesis.^66^ A further consideration is illustrated by the p.Gly115Asp variant, which represents a nonconservative substitution that is consistently scored as damaging in silico (Table 2) and appears to be structurally disrupting (Supplemental Figure 5); yet, phenotypic data argue against pathogenicity. This emphasizes that computational measures and structure-based assessment are necessary and informative, but not sufficient, to predict the pathogenicity of DHRS3 missense variants.

The final homozygous missense variant p.(Glu244Gln), present in family 5, manifests with an inconsistent pattern of findings that leave its interpretation ambiguous (variant of uncertain significance). The substituted Glu244 residue, although located in an α-helical component of the Rossmann fold, is surface-exposed (Supplemental Figure 5) and not fully conserved in human SDR16C family members (Figure 4B^40^). Likely related to this, this variant received relatively lower pathogenicity scores (Table 2). However, both in vivo and vitro measurements of RA revealed significant elevation, supporting that the p.(Glu244Gln) substitution does markedly reduce enzyme activity, possibly by disrupting a (currently undefined) interaction with a partner protein. The craniofacial phenotype of the affected individual (III-1) in family 5 is notably more severe compared with those described in association with homozygous 5′-UTR and p.(Val171Met) variants, with bilateral orofacial clefting and severe hypertelorism, but not craniosynostosis. The presence of scoliosis overlaps with patients in families 1 and 4, but other discordant features include the lack of cardiac malformations and the presence of severe brain malformation (schizencephaly) and severe intellectual disability. Without evidence from additional individuals homozygous at p.(Glu244Gln), we are unable to determine whether this patient has a severe manifestation of DHRS3 deficiency or alternatively has a blended phenotype owing to additional undefined pathology (for example, related to 1 or more of the candidate homozygous variants listed in Supplemental Table 5). Given the unequivocal biochemical evidence that DHRS3 function is compromised both in vivo (Figure 5^35^^,^^44^) and in vitro (Figures 6, Supplemental Figures 10 and 11), we consider it unlikely that the variant is coincidental to the patient’s phenotype.

DHRS3 deficiency joins 2 previously reported recessively inherited clinical disorders that are attributable to RA excess (Figure 1). Deficiency of *CYP26B1* (HGNC:20581), encoding 1 of the 3 cytochrome P450-dependent enzymes that are key for RA catabolism, was previously reported in 14 individuals (4 with fetal demise) with the disorder termed radiohumeral fusions with other skeletal and craniofacial anomalies (RHFCA; MIM 614416).^67^ This comprises a widely variable spectrum of craniofacial malformations, including craniosynostosis, cranial ossification defects with occipital encephaloceles, characteristic facial dysmorphism, joint contractures and fusions, and digit abnormalities, including long slender digits and oligodactyly.^67^, ^68^, ^69^, ^70^, ^71^, ^72^ Although no measurements of RA in patients have been reported, excess embryonic RA-mediated signaling was demonstrated in *Cyp26b1*^*−/−*^ mice.^73^ It has been demonstrated that RA represses proliferation and enhances osteogenic differentiation of suture-derived mesenchymal cells.^74^ This implies that retinoid exposure can promote premature cranial suture fusion via enhanced osteogenesis. In further support of this model, both the hypomorphic *cyp26b1* zebrafish mutant *stocksteif* (*sst*) and zebrafish treated with RA shortly before suture formation exhibit premature synostosis (fusion) of the coronal suture through accelerated osteoblast to (pre-) osteocyte transition.^67^

Antley-Bixler syndrome with genital anomalies and disordered steroidogenesis (ABS1; OMIM 201750) is caused by biallelic variants in the *POR* gene (HGNC:9208) encoding cytochrome P450 oxidoreductase, the universal electron donor for all microsomal P450 enzymes, including CYP26B1.^75^ ABS1 is a multisystem disorder that presents with craniosynostosis, elbow anklyosis, and other joint contractures and genital anomalies among its core clinical features. Analysis of RA levels in both patients and mouse models supports that the skeletal features of ABS are likely attributable to RA embryopathy.^76^^,^^77^ Similar to our observations for *DHRS3*, in both *CYP26B1* and *POR*-related disorders, the combination of 2 variant alleles in patients predicts a hypomorphic effect rather than complete loss of function, likely because the latter would be lethal. In addition to these disorders attributable to RA excess, a recurrent heterozygous nucleotide substitution of *RARA*, encoding p.(Gly289Arg) in RARα, was recently described in 2 individuals with craniosynostosis.^78^ A reduced transcriptional transactivation response to RA was previously demonstrated for *RARA* constructs encoding p.Gly289Arg in the context of somatically acquired mutation in acute promyelocytic leukemia,^79^ but the pathogenic mechanism in a developmental context requires elucidation.

In summary, we provide evidence that homozygosity for the 5‘UTR (family 1) and p.(Val171Met) (families 3 and 4) variants in DHRS3 together define a novel RA embryopathy of humans, associated with a reproducible phenotype comprising craniosynostosis, cardiac malformations, and scoliosis among the more distinctive features. These features are broadly consistent with phenotypes previously attributed to RA excess (CYP26B1 and POR deficiencies) or perturbed signaling (RARA variant). However, we caution that DHRS3 variants may be damaging without being pathogenic because a threshold of RA level needs to be reached to achieve embryopathic effects. Of note, interaction with environmental availability of retinoids may affect the baseline level of RA present in different individuals; therefore, the threshold for pathogenicity of variants could differ between individuals (gene-environment interaction). Future work should aim to define these correlations between genotype and both metabolic and patient-phenotypes in a wider spectrum of patients and variants as further individuals with potential DHRS3 deficiency are identified.

## Data Availability

Genome sequencing data from family 1 (III-2) have been deposited in EGA under accession number EGAS00001007575. Exome sequencing data are not available, owing to ethical restrictions associated with the respective investigations. Variant information has been submitted to ClinVar under accession numbers SCV005685119-SCV005685123.

## ORCID

Andrew Wilkie: http://orcid.org/0000-0002-2972-5481

## Web Resources

CADD, https://cadd.gs.washington.edu/

European Genome-phenome Archive (EGA), https://ega-archive.org/

GeneMatcher, https://genematcher.org/

gnomAD, https://gnomad.broadinstitute.org/

GTEx, https://gtexportal.org/

ImageJ 1.54f, http://imagej.org

Missense3D, http://missense3d.bc.ic.ac.uk/

OMIM, https://omim.org/

REVEL, https://sites.google.com/site/revelgenomics/

UCSC Genome Browser, https://genome.ucsc.edu/index.html

## Conflict of Interest

The authors declare no conflicts of interest.
